# Bonding Strength of a Glass Microfluidic Device Fabricated by Femtosecond Laser Micromachining and Direct Welding

**DOI:** 10.3390/mi9120639

**Published:** 2018-12-03

**Authors:** Sungil Kim, Jeongtae Kim, Yeun-Ho Joung, Jiyeon Choi, Chiwan Koo

**Affiliations:** 1 Department of Electronics and Control Engineering, Hanbat National University, Daejeon 34158, Korea; sung1@hanbat.ac.kr (S.K.); Jeotae@daum.net (J.K.); Yeunho@gmail.com (Y.-H.J.); 2 Department of Laser and Electron Beam Application, Korea Institute of Machinery and Materials, Daejeon 34103, Korea

**Keywords:** microfluidic, femtosecond laser, rapid fabrication, glass welding, bonding strength

## Abstract

We present a rapid and highly reliable glass (fused silica) microfluidic device fabrication process using various laser processes, including maskless microchannel formation and packaging. Femtosecond laser assisted selective etching was adopted to pattern microfluidic channels on a glass substrate and direct welding was applied for local melting of the glass interface in the vicinity of the microchannels. To pattern channels, a pulse energy of 10 μJ was used with a scanning speed of 100 mm/s at a pulse repetition rate of 500 kHz. After 20–30 min of etching in hydrofluoric acid (HF), the glass was welded with a pulse energy of 2.7 μJ and a speed of 20 mm/s. The developed process was as simple as drawing, but powerful enough to reduce the entire production time to an hour. To investigate the welding strength of the fabricated glass device, we increased the hydraulic pressure inside the microchannel of the glass device integrated into a custom-built pressure measurement system and monitored the internal pressure. The glass device showed extremely reliable bonding by enduring internal pressure up to at least 1.4 MPa without any leakage or breakage. The measured pressure is 3.5-fold higher than the maximum internal pressure of the conventional polydimethylsiloxane (PDMS)–glass or PDMS–PDMS bonding. The demonstrated laser process can be applied to produce a new class of glass devices with reliability in a high pressure environment, which cannot be achieved by PDMS devices or ultraviolet (UV) glued glass devices.

## 1. Introduction

Microfluidic devices (or lab-on-a-chip) have been actively researched because they are able to provide rapid reaction and high-throughput screening of very small samples such as protein, DNA, cells, and tissues [1]. The material most commonly used for fabricating microfluidic devices is polydimethylsiloxane (PDMS) because it is transparent, biocompatible, and easy to fabricate. Glass-based microfluidic devices possess many advantages such as high transparency in the visible spectral range, high thermal resistance, and higher mechanical and chemical stability than PDMS [2]. Glass is biocompatible and does not absorb any organic compounds [3]. Glass micromachining, however, requires long fabrication time and efforts. To fabricate micro patterns on a glass sheet, there are several methods, such as wet/dry etching, mechanical fabrication, molding process, the use of photosensitive glass, and so on [4]. Those processes are complex and consist of multiple steps. Furthermore, they require special tools or a clean room facility. In addition, glass-to-glass bonding, such as thermal, fusion, anodic, and adhesive bonding, have disadvantages of long fabrication time and high cost [5]. Moreover, high temperature, high pressure, and high voltage conditions during these types of bonding do not provide an appropriate environment for devices with integrated electrical and mechanical parts [6]. Adhesive bonding using epoxy or ultraviolet (UV) cured glue is very simple, but most adhesives are vulnerable to solvent degradation of the bonding strength, and hence it is difficult to encapsulate devices [7] 

Femtosecond laser processing of glass has attracted huge attention in recent years for applications utilizing glass such as photonics, displays, and optoelectronics [8,9]. It has revolutionized glass micromachining and glass-to-glass bonding to allow simple, rapid, and stable fabrication and packaging processes [10,11,12,13,14,15,16,17,18,19]. Femtosecond laser processing has also reduced the fabrication complexity as it can be utilized for both the formation of micro-patterns and the packaging of a glass chip. The fundamental mechanism of the femtosecond laser glass processing is to induce nonlinear absorption allowing direct photo-ionization in the glass, which is available only at very high laser intensity over a range of ~TW/cm^2^. In this regime, transparent media can directly absorb photons, leading to local melting or structural modification at the exposed area. In particular, femtosecond laser direct welding of glass is one of the most innovative applications of femtosecond laser processing as there is no need to use sacrificial media or indirect heat transfer from intermediate absorbing layers. In most conventional transparent laser welding processes, it is crucial to have interfacial layers between glass substrates that are absorptive at the laser wavelength to absorb photons and deliver heat to the glass substrates [18]. Therefore, conventional transparent laser welding is not direct bonding of glass substrates or a single step process. 

The first glass-to-glass welding by a femtosecond laser was reported by Tamaki et al. in 2005 [11]. They demonstrated the disappearance of Newton’s ring at the femtosecond laser scanned area, which implies the removal of the gap between glass substrates. Thanks to intensive investigations, including those from Cvecek et al. and Okamoto et al., this method achieved higher throughput and process reliability by introducing efficient heat accumulation at MHz repetition, as well as optimized contact treatment methods [12,13,14,15,16,17,18,20,21].

Femtosecond laser glass welding provides several advantages for microfluidic device packaging. First, it has excellent chemical resistance due to the bonding of the base material, which is locally melted without any surface treatment or sacrificial layers [16]. Second, the width of the welding seam is typically a few tens of micrometers, keeping the welded lines as close as possible in a micrometer scale to the microfluidic channel, thus extending the useful space of the substrate [16]. Third, it has higher bonding strength than other bonding methods [16,18,20,21]. Although there is a lack of standardization and evaluation protocol of welding strength measurements thus far, a number of prior studies have already proven that femtosecond laser direct welding shows superior bonding strength compared with conventional bonding methods (e.g., shear stress measurement, three-point bending test, fracture strength, and simple leak check) [19,21,22,23,24].

To fabricate micro-patterns on a fused silica substrate, femtosecond laser assisted selective etching is presented [9,10,24,25,26,27,28]. Laser assisted selective etching is more effective for glass micromachining than a direct ablation process, because the former provides better surface roughness by minimizing debris and cracks. Laser-irradiated parts have a relatively high etch rate due to the laser induced material modification resulting in material density or phase changes, as well as the generation of nano-sized cracks increasing the surface area when immersed in etching fluids such as hydrofluoric acid (HF) and potassium hydroxide (KOH) [25,26]. The fabrication resolution is dependent on the configuration of the beam focusing optics and etching conditions. However, a few micron resolution is readily achievable for typical laser assisted selective etching. 

In recent years, glass microfluidic device fabrication using a femtosecond laser has been actively researched. In particular, it has advantages in terms of fabrication compared with the conventional processing methods, including MEMS and photolithography. Although these conventional techniques have been well established, they are optimized for 2D planer surface microfabrication, while the laser process is able to fabricate a 3D microfluidic structure inside the glass without multiple complex process steps [27,28]. Furthermore, laser patterning is a direct-write scheme that can remove the need for a mask to transfer desired patterns onto the glass. It brings a huge benefit in terms of reducing manufacturing complexity and cost. However, long development time of laser exposed glass chips due to the low etch rate in KOH may be a drawback of the monolithic femtosecond laser fabrication of 3D internal microfluidic channels of glass. According to a prior study by J. Gottmann, the maximum anisotropic etch rate of femtosecond laser exposed glass is typically about 300 μm/h [26]. This implies that the appropriate use of the combination of 2D planar structures with laser direct welding may be reasonable for manufacturing embedded 3D structures in practical applications. 

In this notion, we present a rapid and reliable glass microfluidic device fabrication method for a simple 2D planer channel structure using femtosecond laser assisted selective etching with HF and direct welding by optimizing laser parameters. After fabricating a simple microfluidic device, the endurable pressure of the device was measured to characterize the glass-to-glass welding efficiency. To measure the pressure in a microfluidic channel, a customized hydraulic pressure measurement system was developed and the measured endurable pressure data was compared with that of other bonding methods. In addition, as a demonstration of the application of our proposed method, a droplet generator was fabricated using our method in this work.

## 2. Materials and Methods

### 2.1. Fabrication Procedure

**Figure 1:**
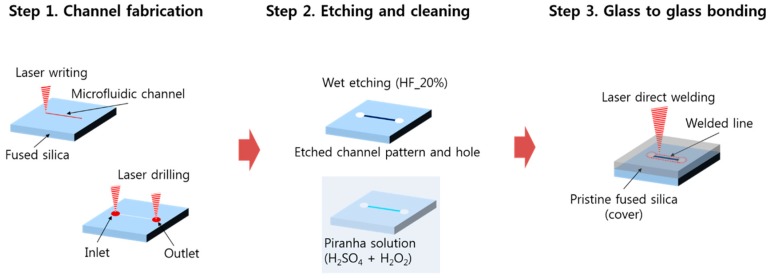
Illustration of the rapid and mask-less glass microfluidic channel device fabrication procedure by using a femtosecond laser. HF—hydrofluoric acid.

We propose a rapid and highly reliable fabrication method for glass microfluidic devices that consists of three steps, as shown in Figure 1. In the first step, a microfluidic channel pattern was directly laser written onto a fused silica glass substrate (JMC glass, Ansan, Korea) with a size of 25 mm × 25 mm × 1 mm and a surface roughness (Ra) of 25 nm. A Yb-doped femtosecond laser amplifier (SatsumaHP2, Amplitude systèmes, Pessac, France) was coupled with a Galvano scanner (IntelliSCAN III, ScanLab, Puchheim, Germany), which was then focused by a f-theta lens (focal length 100 mm, Sill Optics, Wendelstein, Germany). The center wavelength of the laser was 1030 nm and the laser beam profile was Gaussian with a beam quality M^2^ of 1.2. The pulse duration was adjustable from 300 fs to 10 ps. In this step, the pulse duration was kept at the shortest (~300 fs) period to maximize the laser intensity at the used pulse energy of 10 μJ. The pulse energy was sufficient to modify the structure of the glass. The laser beam scanning speed was 100 mm/s at a pulse repetition rate of 500 kHz. The focused laser beam size was about 20 μm and the laser writing was repeated 30 times with 10 μm pitch in the lateral direction to make the width of the microfluidic channel equal to 300 μm. Two holes for inlet and outlet of the microfluidic channel were then laser drilled with a scan speed of 2 m/s. The diameter of the holes was 1 mm. 

In the second step, the laser exposed fused silica substrate was etched in 20% HF acid for 30 min to obtain the microchannel pattern and clear inlet/outlet holes. After the etch process, the substrate was rinsed with DI water and was immersed in a 3:1 mixture of concentrated sulfuric acid (H_2_SO_4_) with hydrogen peroxide (H_2_O_2_), known as a piranha solution. This cleaning procedure is required for the next step to create highly hydrophilic surfaces and to provide optical contact [29] for better laser welding quality. Optical contact is not a mandatory condition for laser welding [30], however, we opted for optical contact for more rigid bonding. In the third step, another glass substrate was placed on top of the etched glass with patterns and they were bonded by femtosecond laser direct welding.

**Figure 2:**
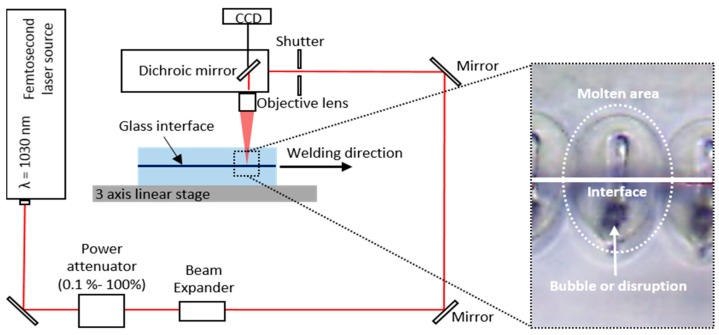
Schematic of laser direct welding experiment setup. The focal position is placed slightly below the interface of two glass substrates and bubbles or disruptions are formed at the position (as shown in the dotted box).

Figure 2 shows the femtosecond laser direct welding setup. Direct welding was performed by inducing heat accumulation at a high repetition rate of 2 MHz in the focused volume using an objective lens 20× (378-867-5, Mitutoyo, Kawasaki, Japan) with a numerical aperture of 0.40. The advantage of high repetition rate (MHz) ultrafast laser irradiation is that the molten layer is uniformly formed and thus bonding stability and strength increase [12]. The focal position is placed slightly below the interface of the two glass substrates. We used a 3D motorized machining stage to translate the glass substrates mounted on a custom tilting jig that compensates for the flatness within 5 μm. The laser pulse energy and welding speed were 2.7 μJ and 20 mm/s, respectively. 

### 2.2. Characterization of the Fabricated Glass Microfluidic Device

To investigate the reliability of the proposed rapid glass microfluidic device packaging method, we performed internal pressure measurement and a leakage test. In addition, a simple droplet generator was fabricated with glass sheets and tested as a demonstration of our fabrication method on microfluidic applications. 

#### 2.2.1. Measurement of Internal Pressure and Leakage Test 

**Figure 3:**
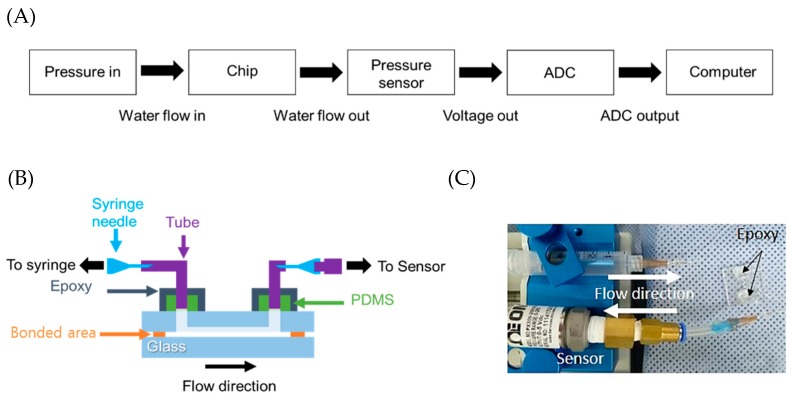
(**A**) Block diagram of the internal pressure measurement test procedure. (**B**) Configuration of interface between chip and peripheral devices and (**C**) the internal pressure measurement system. polydimethylsiloxane (PDMS) blocks were used as an interfacing material between tubing and the glass microfluidic device. Epoxy was applied around the PDMS block and tubing connection area. ADC—analogue to digital.

**Figure 4:**
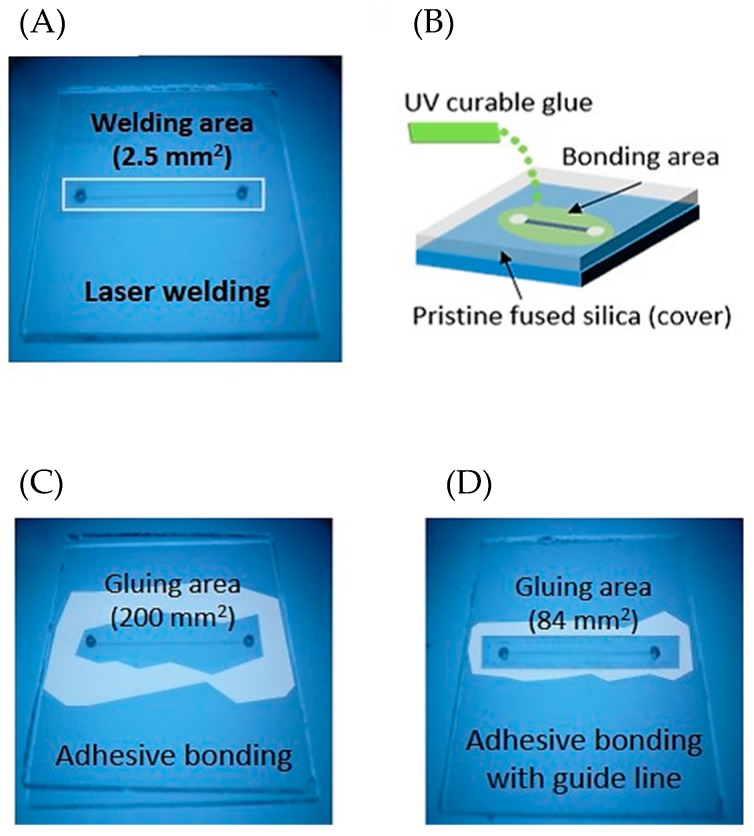
(**A**) Glass microfluidic device welded using the laser. The welding line (white line) was found around the microchannel. (**B**) Schematic of the ultraviolet (UV) curable glue application to bond two glass sheets. (**C**,**D**) Glass microfluidic devices bonded using UV curable glue. The left one had larger gluing area (200 mm^2^) than the right (84 mm^2^).

To measure the endurable pressure of the fabricated glass microfluidic device packaging, we configured an internal pressure measurement system (Figure 3A). While injecting water into the inlet of the microfluidic channel and measuring the pressure at the outlet of the channel, the internal pressure built up until it reached the maximum pressure at which the welding of the glass device was cracked. The internal pressure was continuously measured as a voltage signal by an analogue voltage output pneumatic/fluidic pressure sensor (PX309-200G5V, OMEGA™, Sunbury, OH, USA) and the signal was converted from analogue to digital (ADC) and transferred to a computer. A syringe pump (Fusion 200, Chemyx Inc., Stafford, TX, USA) and a plastic syringe filled with water were used to inject water into the microfluidic channel. The connections between the syringe and the microfluidic channel and between the microfluidic channel and the pressure sensor were made with tubing (Figure 3B). To interface the glass microfluidic device with tubing, PDMS (Sylgard 184, Dow Corning, Midland, MI, USA) blocks were fabricated and bonded to the surface of the glass microfluidic device using oxygen plasma treatment [31]. The epoxy was applied to hold tubing tightly and prevent leakage between it and the PDMS blocks (Figure 3C). In addition, we fabricated glass microfluidic devices bonded using an adhesive glue to compare the bonding strength of our packaging method with that of gluing. While other glass-to-glass bonding methods use entire surface bonding or large bonding area [32], our packaging uses only the small welding area around the patterned microfluidic channel (Figure 4A).

Similarly, UV curable glue is able to be applied on a small bonding area and takes short amount of time to bond two materials, so the glue (Loctite3321, Loctite, Dusseldorf, Germany) was selected for comparison with our packaging. 

The glue was manually applied around the microchannel and a clean glass substrate was placed on it. We then waited until the glue stopped spreading out at the glass interface and exposed it to UV light (Figure 4B). Figure 4C shows the glass microfluidic device bonded with UV glue. Applying the glue on a glass substrate with microchannels was not simple and it was necessary to apply a moderate amount of glue at the proper place. Otherwise, the direction of the glue’s spreading at the glass interface was not controlled and the bonding area was not uniform. From time to time, the glue went into microchannels and blocked the channel. When we used a small amount of glue to prevent the blockage, the bonding strength was weak and the two glass substrates easily came apart. Therefore, another glass microfluidic device was fabricated with a glue guide trench around the microfluidic channel (Figure 4D). The UV glue was applied in the guide line and it was guided well into the trench, not into the microfluidic channel. After fabricating the glass microfluidic devices bonded using the UV curable glue, their bonding strength was tested with an internal pressure measurement system. 

#### 2.2.2. Droplet Generator Experiment

To demonstrate our proposed rapid glass microfluidic device fabrication, we chose a droplet generator with a cross junction (focused flow), because it is widely used in many fields for generating highly reproducible micro- or nano-droplets of water, oil, and other materials [33]. A cross junction for generating droplets and a large chamber for collecting droplets were designed and fabricated on a glass substrate. For making the surface of the microfluidic channel hydrophobic, a water repellent agent (47100, Aquapel, Pittsburgh, PA, USA) was coated. To make water droplets, water mixed with blue dye was injected through the mid inlet, and oil mixed with a surfactant at a 2 wt.% ratio was injected through two side inlets.

## 3. Results and Discussion

### 3.1. Fabrication of the Glass Microfluidic Device 

**Figure 5:**
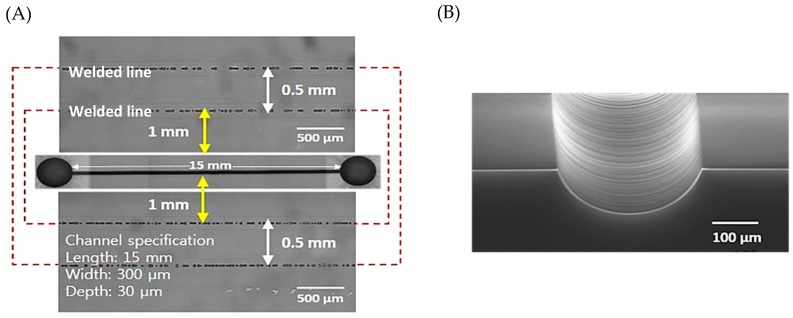
(**A**) Top view of glass microfluidic device using laser assisted selective etching (LASE) and welding. Channel specifications: length 15 mm, width 300 μm, and depth 30 μm. Red dash line welding area. (**B**) Scanning electron microscope (SEM) image showing the formed microfluidic channel with a surface roughness of 360 nm (Ra).

**Figure 6:**
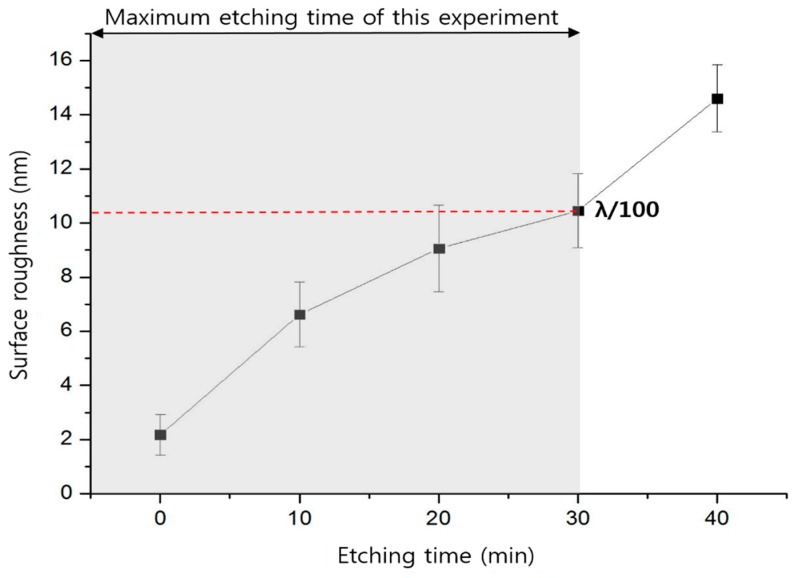
Effect of etching time on the surface roughness of the glass substrate (fused silica) (5 sections, the size of the examined area is 30 × 30 µm^2^).

A simple microfluidic channel was patterned on a fused silica glass substrate and successfully bonded with another fused silica glass substrate. Figure 5A shows the welding seams around the microfluidic channel. The glass substrates were welded at about 500 μm intervals and the width of the welding seam line was 150 μm. The dark circular spots in Figure 5A are bubbles or disruptions of fused silica substrates [34]. A microfluidic channel 15 mm long and 300 μm wide was fabricated using laser assisted selective etching and scanning electron microscope (S-4800, Hitachi, Tokyo, Japan) images were observed, as shown Figure 5B. The surface of the channel was smooth because of the HF wet etch and the cross-sectional shape of the channel was a rounded channel, which is difficult to achieve by normal microfluidic channel fabrication methods such as soft lithography. The inlet/outlet area showed a smooth surface without glass particles. The roughness of the microfluidic channel surface and non-patterned glass surface depended on the time of HF wet etch. If the etch time was less than 10 min, the microfluidic channel surface was still rough to be used as a microfluidic channel, and thus the minimum etch time should be longer than 20 min. When it was etched over 60 min, the glass etch rate dropped dramatically, and hence a long etch time was not necessary. Thus, the etch time was optimized; 30 min was selected and we obtained 360 nm (Ra) roughness for the patterned microfluidic channel. During the wet etch, the non-patterned glass surface is also exposed to the etchant and the surface roughness may affect the welding quality and efficiency. Therefore, we compared the surface roughness using an atomic force microscope (XE-100, Park systems, Gyeonggi-do, Korea) before and after the HF wet etch. Figure 6 shows the measurement results of the surface roughness near the welding area by changing the etching time up to 40 min. According to published papers, optical contacted fused silica welding with ultrafast laser can bridge a 1 to 3 μm gap [20,35]. In conclusion, our etching time of 30 min, generating a surface roughness of 10 nm, which is still less than a hundredth of the wavelength of the welding laser, will not affect the welding efficiency [30].

### 3.2. Internal Pressure Measurement 

To confirm the reliability of the laser welding on a glass microfluidic device, internal pressure measurement was conducted. Upon slowly injecting water into the microfluidic channel in the glass device, the internal pressure was built up at a rate of 100 kPa/min and suddenly dropped after a point of time. The highest pressure was 1.4 MPa. However, the glass-to-glass welding still remained, but the epoxy sealing on the tubing connection between the glass device and the tubing was broken. Therefore, we carried out a tensile test (Instron 5848 Instron, Norwood, MA, USA) and the measured value was 7.5 MPa. The glass microfluidic device with an 84 mm^2^ gluing area could prevent blockage of the microchannel by the UV glue with help of a UV glue guide line around the microchannel, but the bonding was broken at 1.1 MPa internal pressure. The glass device with a larger gluing area (200 mm^2^) was able to withstand internal pressure of at least 1.4 MPa. Because of failure of the tubing connection, higher pressures could not be tested. The UV glue bonding method is able to provide a strong bond of two glass substrates, but it has disadvantages of requiring a large area of UV glue at the interface of the two glass substrates and an additional process for fabricating the UV glue guide line to prevent the applied UV glue from entering the microchannel. In addition, it requires preparation time of about 20 to 30 min for the UV glue to spread as well as UV curing time. Table 1 lists the reported bonding strength of the conventional bonding methods and our bonding method together. The PDMS-to-glass and PDMS-to-PDMS plasma bonding, which are widely used for fabricating microfluidic devices, endured 510 kPa and 551 kPa, respectively, in the liquid injection test [31,36]. The bonding strength of our method was at least two-fold higher that that provided by other methods. The glass-to-glass bonding method using a microwave kiln, removable ceramic paper, and a microwave oven showed about 1 MPa bonding strength in the liquid injection test [37]. Higher pressures could not be applied because of the failure of the tubing connection and it was assumed that their bonding strength is extremely high, corresponding with the glass strength. However, the method requires specific ceramic papers and the microchannel shape was deformed, while the entire bonding area melted and was bonded. The fabrication time for microchannels and bonding in a microwave oven was about 2 to 4 min, but it required a long cooling time, exceeding 45 min. In the cases of anodic bonding at room temperature and glass-to-glass rapid bonding using Pyrex glass, the bonding strength was measured as 29.7 MPa and 2.5 MPa, respectively, with a tensile test [38,39]. When comparing the strength of our method and anodic bonding and the rapid Pyrex glass bonding, the strength of our bonding was lower than that of the anodic bonding, but higher than that of the rapid Pyrex bonding. Except for the anodic bonding method, our laser welding method could endure higher force than other methods. Thus, we believe that the laser welding method provides reliable bonding. In addition, in terms of time consumption, our method was at least 10 times faster than other bonding methods.

### 3.3. Liquid Leakage and Droplet Generator

**Figure 7:**
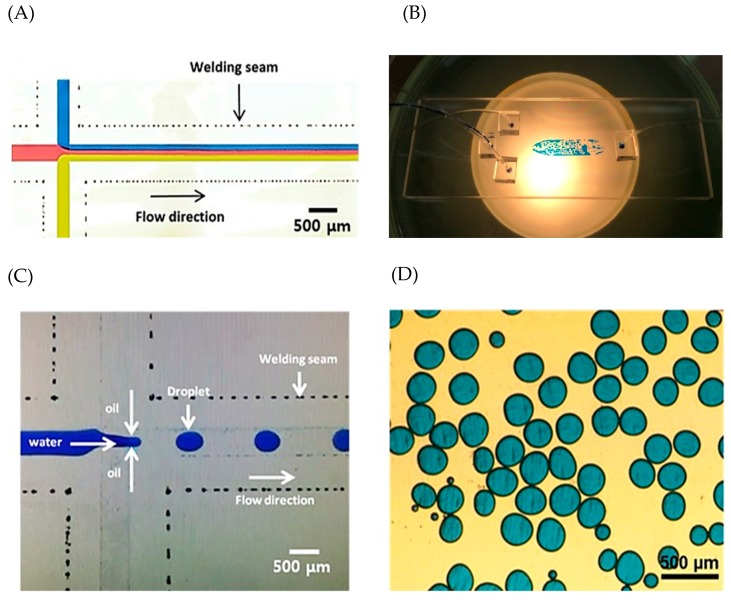
(**A**) Picture of the laminar flow generated at the microfluidic channel fabricated by laser test. (**B**) Picture of the fabricated droplet generator. (**C**) Picture of droplet generating at the cross-section. Blue dye was mixed with water to show the droplets clearly. (**D**) Generated droplets collected at a reservoir.

We fabricated a droplet generator to investigate the possibility of applying our fabrication method to microfluidic devices in different fields. Before the droplet generation test, three dyes were injected into the microfluidic channels to generate a laminar flow and to observe the area of the laser welding seam to check if there was leakage. No leak was observed and a laminar flow was formed successfully (Figure 7A). Therefore, the microchannel surface roughness was smooth, allowing generation of a laminar flow, and the bonding tightness was enough to test for a normal microfluidic test. For the droplet generator test, the water with blue dye was injected through the mid inlet and the oil mixed with the surfactant at 2 wt.% ratio was injected through two side inlets. Micro-droplets were generated sequentially and successfully collected in a reservoir (Figure 7B–D). The developed fabrication method provides not only reliable packaging, but also the capability for application to glass microfluidic devices.

## 4. Conclusions

All glass microfluidic devices have advantages over polymer based microfluidic devices, but they are difficult to fabricate and can only be used for specific experiments. However, in this paper, we propose a rapid and reliable glass microfluidic device fabrication strategy based on laser assisted selective etching and direct welding of simple 2D planar channels, similar to most PDMS devices using optimized laser parameters. It even provides high bonding strength compared with conventional bonding methods. We believe that the fabrication method introduced here has potential to be used for glass microfluidic devices in harsh environments that PDMS microfluidic devices cannot endure. In future work, we will develop a glass microfluidic device using solvents or stains (e.g., Nile red and Rhodamine B) that cannot be applied to PDMS devices and a glass lab-on-a-chip enduring high pressure conditions.

## Figures and Tables

**Table 1:** Comparison of glass bonding method. PDMS—polydimethylsiloxane.

Materials	Bonding Method	Bonding Time (h)	Maximum Bonding Strength (MPa)	Test Method
PDMS–Glass [31]	Plasma	0.5~2	0.51	Pressure injection
PDMS–PDMS [36]	Plasma	0.5~2	0.55	Pressure injection
Glass–Glass [37]	Microwave oven	1	>1 (assume: 1 to 30)	Pressure injection
Glass–Glass [38]	Anodic	24	29.7	Tensile test
Glass–Glass [39]	Pyrex	1.3	2.5	Tensile test
Glass–Glass	UV adhesive	0.5	1.1	Pressure injection
Glass–Glass	Laser welding (this work)	<0.08 (5 min)	>1.4	Pressure injection
			7.5	Tensile test
